# Modification of ALPPS to avoid ischemia and congestion after stage 1: a case report

**DOI:** 10.1186/s40792-022-01490-x

**Published:** 2022-07-22

**Authors:** Mai Ishihara, Yuki Takahashi, Kenichi Matsuo, Akihiro Nakamura, Shinji Togo, Kuniya Tanaka

**Affiliations:** 1 Department of General and Gastroenterological Surgery, Showa University Fujigaoka Hospital, 1-30 Fujigaoka, Aoba-ku, Yokohama, Kanagawa 227-8501 Japan; 2 Ishikawacho Internal Medical Clinic, 1-3-7 Matsukage-cho, Naka-ku, Yokohama, Kanagawa 231-0025 Japan

**Keywords:** Hepatectomy, ALPPS, Modified technique, Ischemia, Congestion

## Abstract

**Background:**

Associating liver partition and portal vein ligation for staged hepatectomy (ALPPS) has been advocated for treating advanced liver tumors, but the devascularized ischemic area resulting from liver parenchymal division can become a nidus for sepsis. We present a patient who underwent ALPPS modified to avoid ischemia and congestion after liver partitioning during stage 1.

**Case presentation:**

ALPPS was carried out for a patient with multiple bilobar liver metastases from rectosigmoid colon cancer. The 2-stage treatment included 3 partial resections within the left lateral section and parenchymal division at the umbilical fissure with right portal vein ligation as stage 1, followed by right trisectionectomy as stage 2. During parenchymal division at the umbilical fissure, Segment 4 portal pedicles and the middle hepatic vein had to be resected at their roots. To safely accomplish this, combined resection of Segment 4 and the drainage area of the middle hepatic vein was performed after parenchymal partition, aiming to avoid ischemia and congestion within the remnant liver. Successful stage 2 hepatectomy followed later. No ischemia or congestion occurred during stage 1 or 2.

**Conclusions:**

During ALPPS, ischemia and congestion after stage 1 must be avoided to reduce morbidity and mortality. The modification described here should reduce likelihood of severe postoperative complications.

## Background

Although associating liver partition and portal vein ligation for staged hepatectomy (ALPPS) has been advocated for treating advanced liver tumors, increased morbidity and mortality remain serious problems. A variety of modifications to reduce adhesions, prevent tumor spread, and avoid devascularization during hepatic division have been undertaken [1]. Recent variations mostly aim to reduce surgical invasiveness and risk of stage 1, but they might make stage 2 more complicated and risky.

Principal causes of mortality in previous reports of ALPPS have been sepsis [2–4] and liver failure [2]. Excessive mortality of ALPPS may be largely attributable to sepsis arising from the devascularized ischemic area resulting from liver parenchymal division [3–6]. Similarly, in living-donor liver transplantation, sepsis can arise from the congested area produced by middle hepatic vein (MHV) interruption in the implanted right liver graft [7]. When sepsis originates in a necrotic focus, the involved tissue must be removed [8], although such an undertaking is difficult in the presence of sepsis and disseminated intravascular coagulopathy (DIC) [3]. Accordingly, avoidance of focal congestion or ischemia that could progress to necrosis is a vital consideration during stage 1 of ALPPS.

Here, we present a patient with multiple colorectal liver metastases who underwent ALPPS including certain modifications of stage 1 aiming to avoid ischemia and congestion after liver partitioning.

## Case presentation

**Fig. 1:**
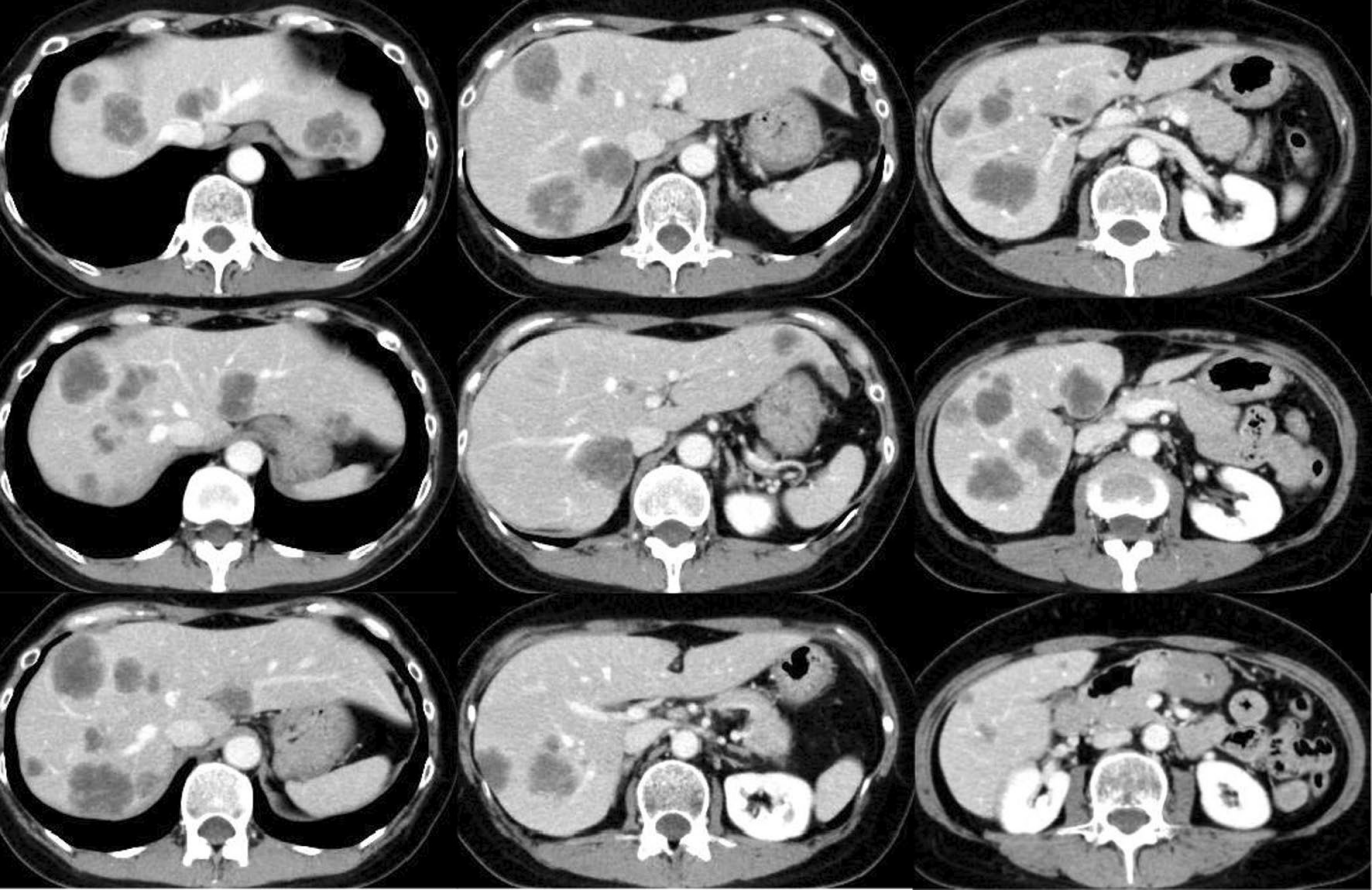
Computed tomographic findings before chemotherapy. Multiple metastases diffusely involve the liver

**Fig. 2:**
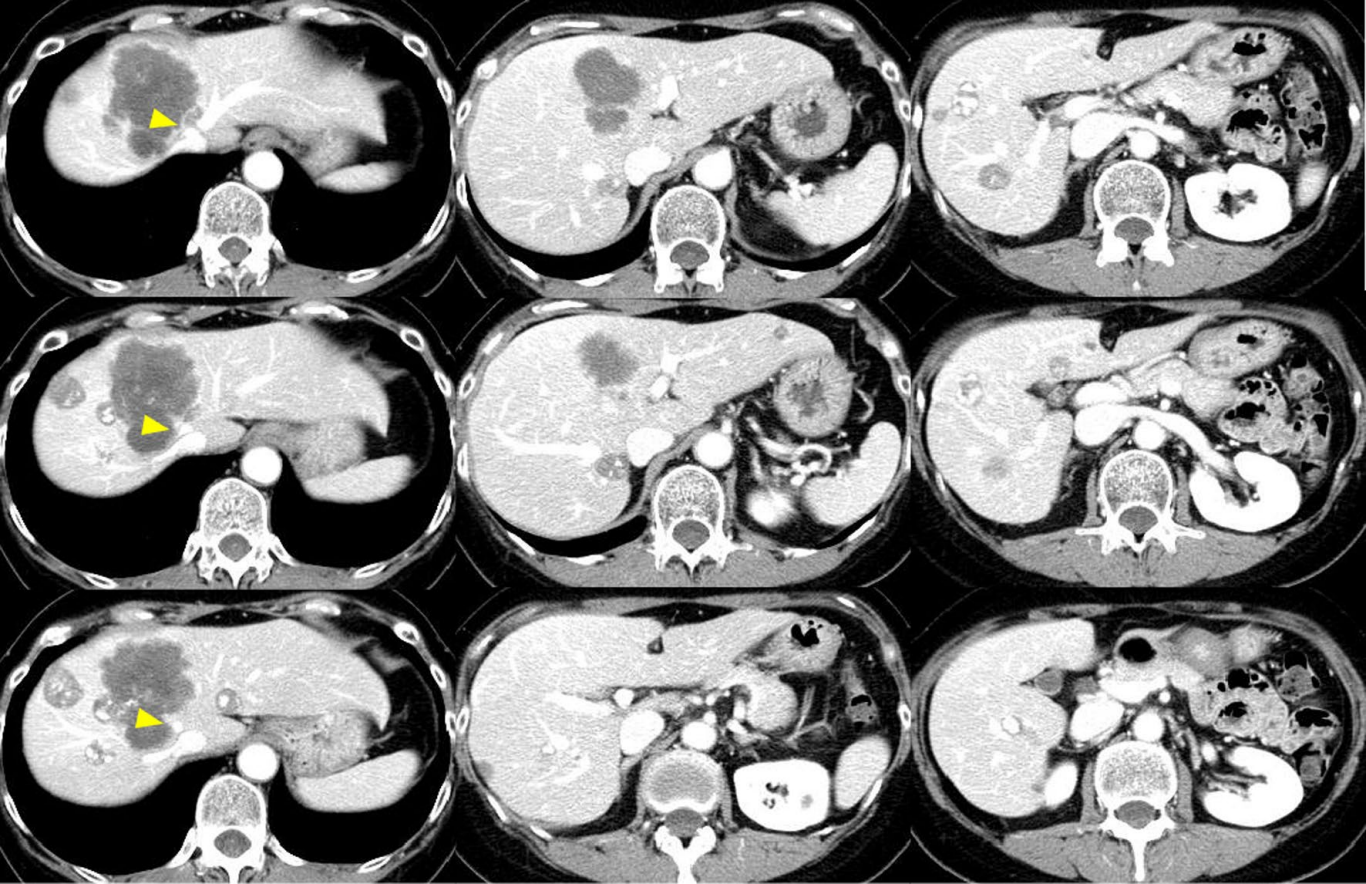
Computed tomographic findings after chemotherapy. While liver metastases are reduced overall, tumor involving segments 4 to 8 has invaded the MHV near the confluence of the MHV and the LHV (arrowhead)

A 61-year-old woman who had undergone laparoscopic high anterior resection for rectosigmoid colon cancer and developed multiple bilobar liver metastases was referred to our institution 2 years and 8 months after surgery for the primary tumor. After liver metastases were detected by computed tomography (CT; Fig. 1), she received 34 cycles of XELOX (capecitabine and oxaliplatin) plus bevacizumab and 2 cycles of FOLFIRI (5-fluorouracil, folinic acid, and irinotecan) plus aflibercept. After chemotherapy, CT (Fig. 2) showed considerable tumor shrinkage except for a metastasis located in segment 4 that had extended to segment 8 invaded the MHV near its confluence with the left hepatic vein (LHV).

When hepatic reserve was assessed at the time of referral to our hospital, the indocyanine green retention rate at 15 min (ICGR-15) was 12.5%, and the rate of ICG disappearance from plasma (KICG) [9] was 0.1386. A 99 mTc-galactosyl serum albumin (GSA)-rectified ICG value obtained using a GSA scintigraphic receptor index according to the following formula, 105–101 × LHL15 (uptake ratio of the liver to that of the liver and heart at 15 min) [10], was 16.12 (Table 1). Serum albumin (3.8 g/dL), total bilirubin (0.5 mg/dL), platelet count (29.6 × 10^4^/mL), and other routine laboratory values were essentially normal.

**Table 1:** Changes in liver volume and hepatic reserve

	Before stage 1	Before stage 2
*Liver volume*		
Planned resection volume	706.2 cm^3^	592.3 cm^3^
	77.5%	59.0%
FLR volume	205.2 cm^3^	411.5 cm^3^
	22.5%	41.0%
*Hepatic reserve*		
ICG R15min	12.5%	10.29%
KICG	0.1386	0.1516
GSA ICG	16.12	13.09
*Prognostic evaluation*		
Prognostic score	62.5	42.8
Rem KICG	0.0312	0.0621

*FLR* future liver remnant, *ICG* indocyanine green, *GSA* 99mTc-galactosyl serum albumin, *Rem KICG* KICG of the future liver remnant. Data before stage 2 were calculated at 11 days after stage 1

Removal of all 19 metastatic tumors required right trisectionectomy with partial resections of the left lateral section. Estimated resection volume, resection volume relative to total liver volume, and planned remnant liver volume, respectively, were 706.2 cm^3^, 77.5%, and 205.2 cm^3^ (Table 1). Prediction scores [11] for the planned procedure, calculated using the formula, -84.6 + 0.933 a + 1.11 b + 0.999 c with a as anticipated resection fraction (%), b as ICGR-15 (%), and c as patient age in years, were 62.5 using ICGR-15 and 66.5 using GSA-rectified ICG. Further, KICG for the future liver remnant (remKICG), calculated using the formula, KICG × % volume of the future liver remnant (FLR) /100 [12], was 0.0312 (Table 1). Both a prediction score over 55 and a remKICG below 0.05 indicated that any adequate procedure performed as a single stage would not be tolerated based on FLR function. We therefore chose to perform ALPPS.

**Fig. 3:**
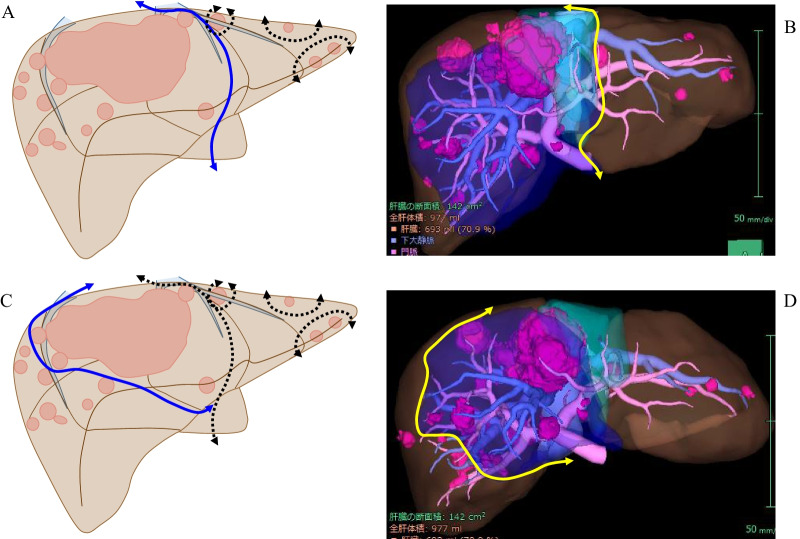
ALPPS procedure in the present patient. Panel **a** shows the scheme of parenchymal division at the umbilical fissure (blue line) following 3 partial resections within the left lateral section (black dotted line). Panel **b** shows territories of S4 portal pedicles (green area) and MHV (blue area) as depicted by 3-dimensional (3D) computed tomography. The resection line at the left umbilical fissure is shown in yellow. Panel **c** shows an additional line of resection to excise the territories of both S4 and the MHV (in blue). Dotted lines indicate 3 partial resections and the resection line at the umbilical fissure. Panel **d** shows a 3D-CT image with the line for additional resection of S4 and the MHV territory in yellow

**Fig. 4:**
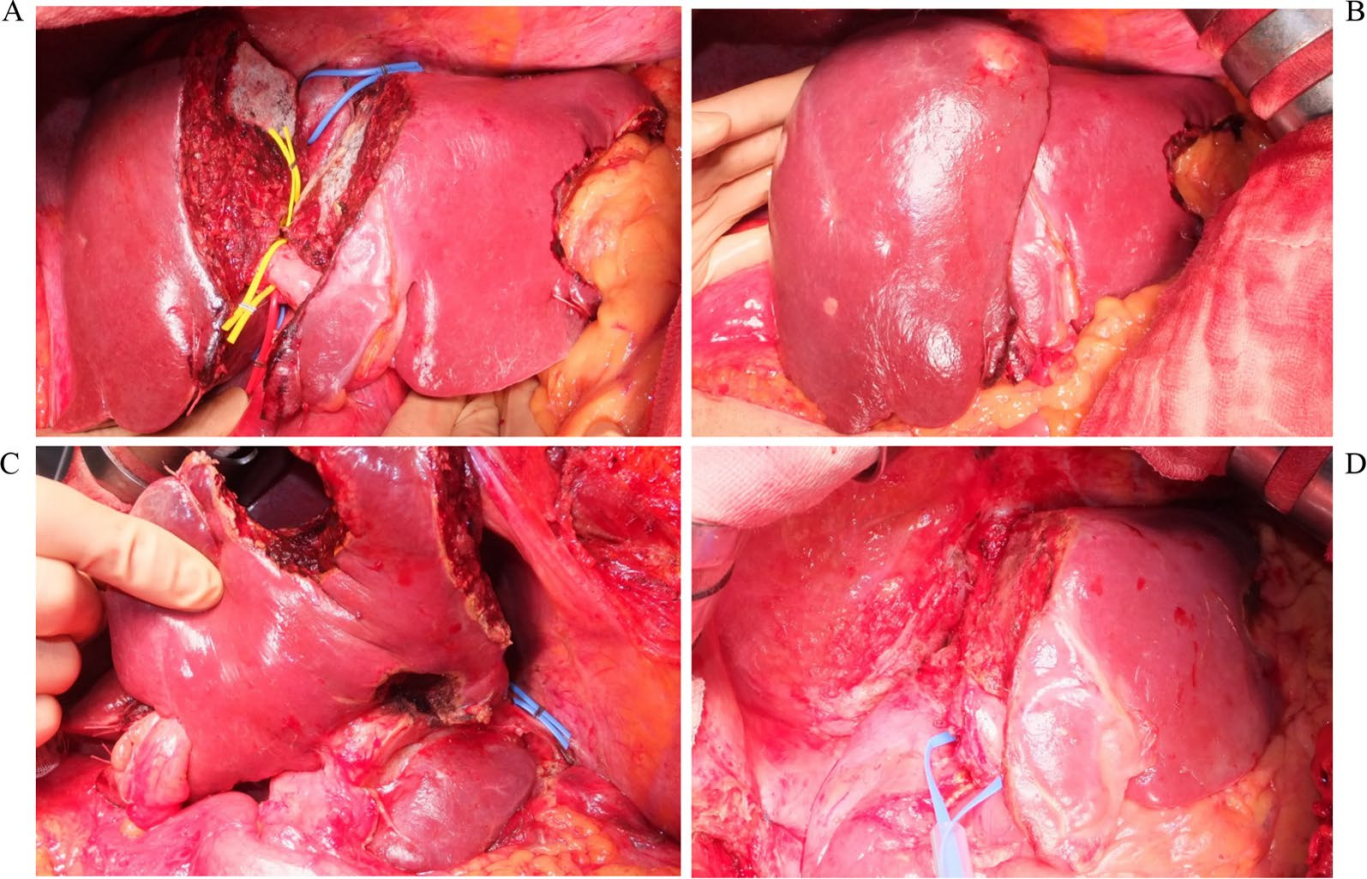
Intraoperative findings at stages 1 and 2. Panels **a** to **c** show liver status at the conclusion of stage 1. Panel **d** shows status after right trisectionectomy at stage 2

**Fig. 5:**
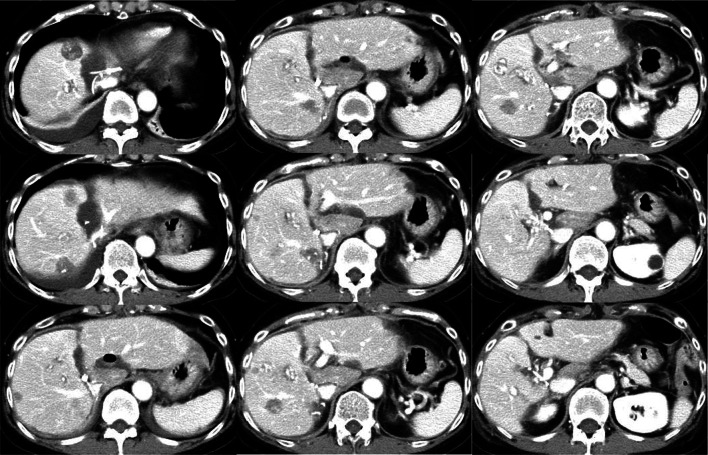
Computed tomographic findings preceding stage 2 of ALPPS. No ischemia or congestion is evident, although blood supply appears slightly decreased within the left caudate lobe

Initially we planned to perform classical ALPPS consisting of 3 partial resections within the left lateral section and parenchymal division at the umbilical fissure with right portal vein ligation in stage 1, followed by right trisectionectomy in stage 2. This procedure would require division of portal pedicles in segment (S) 4, which risks parenchymal ischemia and necrosis in the S4 territory. Further, this patient’s tumor already had invaded the MHV; subsequent tumor extension to the common channel of the LHV and the MHV likely would occur during the interval between stages 1 and 2, interfering with completion of the procedure and likely requiring division of the root of the MHV to the common channel. When the MHV is divided at the confluence of the common channel, parenchymal congestion within the MHV territory becomes likely. We therefore removed S4 together with the MHV territory following parenchymal division at the left umbilical fissure. After determining the border of the territory between the MHV and the right hepatic vein (RHV) by simultaneous occlusion of both the right hepatic artery and the root of the RHV, we removed S4 and the MHV territory (Fig. 3; Fig. 4A–C). Eleven days after stage 1, FLR volume and remKICG had increased to 411.5 cm^3^ and 0.0621; the prediction score was reduced to 42.8 (Table 1, Fig. 5). At the time of right trisectionectomy, the required extent of resection had been reduced to the right posterior section with part of the anterior section. This was performed successfully at 14 days after stage 1 without occurrence of morbidity (Fig. 4D).

## Discussion

According to the original ALPPS procedures reported in 2012, both the S4 portal pedicles and the MHV would be divided during parenchymal resection at the left umbilical fissure [13]. This sometimes resulted in severe ischemia and congestion creating a nidus for sepsis. Such necrosis of S4 frequently occurs following parenchymal transection at the level of the falciform ligament with division of all S4 portal pedicles [3, 13, 14]. Abscess development within this ischemic S4 deprived of both arterial and portal inflow after stage 1 has been reported to lead to sepsis, multi-organ failure, and postoperative mortality [3]. Even though venous devascularization resulting in congestion could help to stimulate rapid, marked FLR hypertrophy [13, 15], some authors point out that congestion also increases risk of ischemia and necrosis after parenchymal division [5, 7]. After living-donor liver transplantation, sepsis can develop similarly, spreading from the congested area produced by MHV interruption in implanted right liver graft [7]. In ALPPS, preservation of outflow structures generally is considered important for avoiding parenchymal necrosis after liver partition. A report from the first international expert meeting on ALPPS emphasized the need to preserve venous drainage to complement arterial inflow to the deportalized area of the liver [16].

Various techniques have been proposed to minimize ischemia and congestion following parenchymal division. Partial ALPPS represents an attempt to decrease invasiveness and improve safety of stage 1 [17]. According to the first report of partial parenchymal transection, its main purpose was to preserve outflow via the hepatic vein. Additionally, partial partition should spare some branches of the portal pedicles, avoiding the dangers of parenchymal ischemia. We previously reported an alternate modified ALPPS procedure including portal pedicle preservation during parenchymal division in order to avoid necrosis while still providing sufficient stimulation of rapid hypertrophy after stage 1. However, this procedure is somewhat difficult to perform [18]. Some other surgeons advocated routine additional resection of S4, which became a potential source of morbidity in the original ALPPS procedure [5].

As outlined above, 3 options for avoiding ischemia and congestion include partial ALPPS, modified ALPPS preserving S4 portal pedicles and the MHV during parenchymal division, and additional removal of the MHV drainage area including S4. In our patient, prehepatectomy chemotherapy had been ineffective against the metastasis located in S4, where the tumor had invaded the MHV and threatened to involve the common trunk of the MHV and the LHV. Tumor invasion of the common trunk would preclude continuing with stage 2 unless a complex procedure combining resection and reconstruction of the LHV with removal of right trisections was performed. To prevent further invasion of the hepatic vein by the S4 tumor as well as ischemia and congestion, we resected the territories of both the S4 portal pedicles and the MHV in the course of parenchymal division at the falciform ligament during stage 1. No ischemia or congestion was demonstrated by CT at 11 days following stage 1 (Fig. 5). The patient experienced no morbidity after either stage 1 or 2.

Among hepatectomy procedures, a transection plane at the left umbilical fissure to the right of the umbilical portion of the left portal vein during right trisectionectomy or to the right of the main portal pedicle involving the right anterior section during extended posterior sectionectomy could induce ischemia in S4 or the lateral portion of the anterior section. Similarly, a transection plane at the left umbilical fissure during right trisectionectomy or to the left of the MHV during right hepatectomy extended to S4 could induce congestion after the confluence of the MHV with the inferior vena cava is ligated and divided. Accordingly, these hepatectomy procedures may represent important indications for our proposed modification.

Considering our ALPPS experience with over 20 patients, the frequency of postoperative complications representing Clavien–Dindo class III or higher has been similar between the original ALPPS procedure and modified ALPPS procedures involving preservation of portal pedicles or hepatic veins near the plane or removal of potentially ischemic or congested areas after parenchymal transection. Such complications followed about 35% of both unmodified and modified ALPPS procedures (data not shown). However, 1 death followed an unmodified ALPPS procedure after sepsis originated in a necrotic focus of ischemic liver parenchyma that developed after parenchymal partitioning [4].

Advantages of parenchyma-sparing resection for colorectal liver resection recently have been reported [19, 20]. However, in the present case, the right hepatic vein was invaded at several points by metastases, restricting options for partial resection combined with resection and reconstruction of the invaded hepatic vein. Further, some viable metastatic nodules no longer visualized in images after multiple cycles of chemotherapy might have been left behind after partial resections for remaining radiologically evident metastases in the right posterior section and lateral portion of the right anterior section. Viable tumor cells reportedly could remain within as many as half of previously demonstrated nodules not apparent following chemotherapy [21–23]. For these reasons, we carried out our modified ALPPS for the present patient. The clinical role of our proposed procedure awaits confirmation by analysis of results in future patients.

## Conclusions

Minimizing likelihood of morbidity and mortality requires avoidance of ischemia and congestion following stage 1 of ALPPS. The modified procedure described here should increase safety for patients undergoing difficult ALPPS.

## Data Availability

Not applicable.

## Abbreviations

ALPPS: Associating liver partition and portal vein ligation for staged hepatectomy
CT: Computed tomography
FLR: Future liver remnant
GSA: Galactosyl serum albumin
ICGR-15: Indocyanine green retention rate at 15 min
KICG: Plasma disappearance rate of indocyanine green
LHV: Left hepatic vein
MHV: Middle hepatic vein
S: Segment
3D-CT: Three-dimensional computed tomography
